# Impact of visual acuity on developing literacy at age 4–5 years: a cohort-nested cross-sectional study

**DOI:** 10.1136/bmjopen-2015-010434

**Published:** 2016-02-16

**Authors:** Alison Bruce, Lesley Fairley, Bette Chambers, John Wright, Trevor A Sheldon

**Affiliations:** 1 Bradford Institute for Health Research, Bradford, UK; 2 Health Sciences, University of York, York, UK; 3 Division of Epidemiology & Biostatistics, University of Leeds, Leeds, UK; 4 Institute for Effective Education, University of York, York, UK; 5 Hull York Medical School, University of York, York, UK

**Keywords:** Vision Screening, Visual Acuity, Child, Acheivement

## Abstract

**Objectives:**

To estimate the prevalence of poor vision in children aged 4–5 years and determine the impact of visual acuity on literacy.

**Design:**

Cross-sectional study linking clinical, epidemiological and education data.

**Setting:**

Schools located in the city of Bradford, UK.

**Participants:**

Prevalence was determined for 11 186 children participating in the Bradford school vision screening programme. Data linkage was undertaken for 5836 Born in Bradford (BiB) birth cohort study children participating both in the Bradford vision screening programme and the BiB Starting Schools Programme. 2025 children had complete data and were included in the multivariable analyses.

**Main outcome measures:**

Visual acuity was measured using a logMAR Crowded Test (higher scores=poorer visual acuity). Literacy measured by Woodcock Reading Mastery Tests-Revised (WRMT-R) subtest: letter identification (standardised).

**Results:**

The mean (SD) presenting visual acuity was 0.14 (0.09) logMAR (range 0.0–1.0). 9% of children had a presenting visual acuity worse than 0.2logMAR (failed vision screening), 4% worse than 0.3logMAR (poor visual acuity) and 2% worse than 0.4logMAR (visually impaired). Unadjusted analysis showed that the literacy score was associated with presenting visual acuity, reducing by 2.4 points for every 1 line (0.10logMAR) reduction in vision (95% CI −3.0 to −1.9). The association of presenting visual acuity with the literacy score remained significant after adjustment for demographic and socioeconomic factors reducing by 1.7 points (95% CI −2.2 to −1.1) for every 1 line reduction in vision.

**Conclusions:**

Prevalence of decreased visual acuity was high compared with other population-based studies. Decreased visual acuity at school entry is associated with reduced literacy. This may have important implications for the children's future educational, health and social outcomes.

**Strengths and limitations of this study**

- Data linkage provides a comprehensive data set which allowed adjustment for confounding factors.
- This is one of the first studies to investigate the impact of reduced vision on educational attainment.
- The study is based in a large multiethnic population.
- The study is limited by its cross-sectional nature.
- Not all participants have complete data sets for all the variables.

## Introduction

The UK National Screening Committee (UK NSC) recommends that vision screening should be provided to all children at age 4–5 years^1^; these recommendations form part of the Healthy Child Programme.^2^ However, the evidence supporting this recommendation is weak. In particular, there are limited data on the prevalence of vision levels in children at age 4–5 years when they first enter school, and the effect of reduced vision on educational attainment in children has not yet been established.^1^ ^3^ Early literacy is a key indicator of future reading performance and educational attainment^4^ ^5^ which in turn affects long-term health and social outcomes.^6^ ^7^ It is intuitive that poor vision will impact on a child's reading ability and lead to educational underachievement, yet there is little evidence to confirm this. At a time of change and uncertainty in the commissioning of vision screening services, it is important to understand both the level of vision in the population and the impact this is likely to have on future health and social outcomes.^8^ ^9^ Better evidence is therefore required to inform child screening policy both in the UK and internationally.

The aim of this study is to determine the prevalence of poor vision in a multiethnic population and explore the impact of reduced vision on developing literacy skills in young children as they start primary school at age 4–5 years.

One of the challenges to the investigation of a causal relationship between vision and literacy is the potential confounding effect of socioeconomic factors. It is well known that socioeconomic deprivation is associated with poor levels of literacy; therefore, any study seeking to explore the degree to which poor vision affects literacy over and above effects of socioeconomic and other demographic factors requires comprehensive data collection.

The city of Bradford in the UK offers the opportunity to conduct such a study because it is the setting for the Born in Bradford (BiB) birth cohort study^10^ which collected detailed epidemiological data during pregnancy, at birth and literacy measures in a subgroup of the children in their first year of school. Bradford also has a comprehensive vision screening programme which provides a detailed profile of children's vision. These data provide the unique opportunity to explore the association between visual acuity (VA) and early developing literacy with adjustment for the effects of potential confounding variables.

## Methods

Vision screening and literacy measures were prospectively collected from children in their first year of primary school within the same school term over two consecutive years (2012–2013 and 2013–2014). Vision screening data from all participants was used to determine the prevalence of poor vision. Baseline epidemiological data collected from mothers and children of the BiB cohort, literacy measures and data captured from the vision screening programme were linked in order to investigate the impact of vision on literacy. Details of each element are provided below.

### Born in Bradford

BiB is a longitudinal multiethnic birth cohort study aiming to examine the impact of environmental, psychological and genetic factors on maternal and child health and well-being.^10^ Bradford is a city with high levels of socioeconomic deprivation and ethnic diversity. Approximately half of the births in the city are to mothers of South Asian origin. Women were recruited while waiting for a glucose tolerance test, routinely offered to all pregnant women registered at the Bradford Royal Infirmary at 26–28 weeks gestation. For those consenting, a baseline questionnaire was completed. The full BiB cohort recruited 12 453 women during 13 776 pregnancies between 2007 and 2010 and the cohort is broadly representative of the city's maternal population.^10^ Ethics approval for the data collection was granted by Bradford Research Ethics Committee (Ref 07/H1302/112).

### Literacy

As part of a separate BiB ‘Starting Schools Programme’ exploring literacy, movement and well-being, children's literacy levels on school entry were measured in school by experienced research assistants. All 123 Bradford primary schools were invited to participate, 76 separate schools agreed to take part and 2929 BiB children received a literacy assessment between September 2012 and July 2014.

Early literacy skills that predict future reading performance include letter identification.^4^ Letter identification measures the child's ability to identify single letters, an essential skill mastered prior to reading and one of the best predictors of reading achievement.^11^ Letter identification was measured using the Woodcock Reading Mastery Tests-Revised (WRMT-R) subtest: letter identification, a validated reading skill test.^12^

In addition, a measure of acquired or receptive vocabulary was recorded using the British Picture Vocabulary Scale (BPVS).^13^ It has been shown to be an important indicator of cognitive ability, providing a representation of the measure of IQ in young children. This measure is included to adjust for potential confounding due to levels of general cognitive ability.

Both measures are standardised taking into account the child's age and time of testing during the academic year, a mean score of 100 would be expected for a given population.^12^ ^13^

### Vision

**Figure 1:**
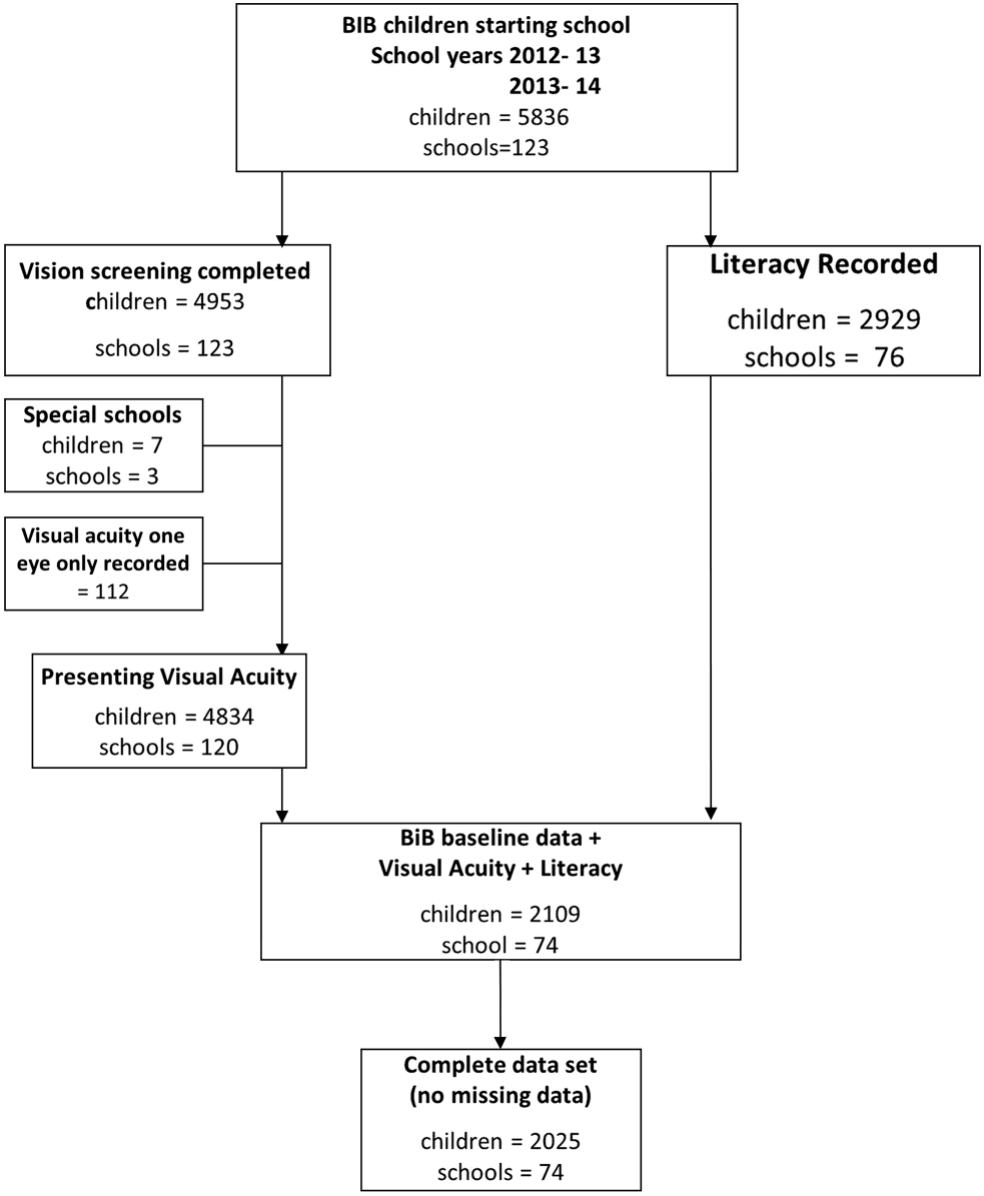
Flow chart of data linked between Bradford vision screening programme, Starting Schools and Born in Bradford (BiB) participants.

A vision screening programme for school children aged 4–5 years has been established in Bradford. The screening programme is conducted in school by orthoptists. Owing to the nature of the programme being conducted after school entry, coverage is high at 97%.^14^ In total, 11 186 children from 123 primary schools across the city participated in the vision screening programme. In total, 5836 BiB children were eligible for the study (started school between September 2012 and July 2014) and 4953 (85%) BiB children had completed the vision screening programme prior to the data linkage (figure 1). The vision screening assessment includes standard protocols for measurement of distance VA^15^ ^16^ right and left eyes, with spectacles if worn. The VA test was administered by orthoptists, performed at a distance of 3 m and VA was measured to threshold (ie, best achievable VA with no defined endpoint). Additional tests carried out by the orthoptists were cover test, ocular motility and non-cycloplegic auto refraction (Welch-Allyn Inc Skaneateles, New York, USA). VA was measured with an age appropriate logMAR Crowded Test (Keeler, Windsor)^15^ which has four letters per line each letter having a score of 0.025; the total score for each line represents 0.1 log unit. A matching card is used and knowledge of letters is not necessary to perform the test. In total, 4834 BiB children completed the vision screening and had VA recorded for both right and left eyes (figure 1). In total, 118/4834 (2%) of children were unable to match letters, they were tested using Kay Pictures Crowded LogMAR (Kay pictures, Tring UK).^17^ ^18^ Refractive error is commonly associated with reduced VA in young children^19^; hence, non-cycloplegic autorefractor readings for the right and left eyes were recorded and a mean spherical equivalent (sphere plus half-negative cylinder) calculated for each eye of individual children.^19^ ^20^ In total, 4578 out of 4834 children had a mean spherical equivalent calculated. Data from the vision screening programme used for the analyses include presenting VA (best VA right or left eye, with glasses if worn) and the mean spherical equivalent from that same eye.

Presenting VA will be referred to as VA for the rest of the paper and in all tables. VA was categorised to examine prevalence of levels of vision. Four categories were established: better than 0.20 logMAR (a pass on visual screening), 0.225–0.30, 0.325–0.40, (referred to as ‘poor vision’ in many published studies)^19^ ^21^ and worse than 0.4 (a category used to define visual impairment by the WHO).^22^ VA was treated as a continuous variable in the statistical modelling allowing for letter-by-letter scoring.

### Statistical analysis

Multilevel regression analysis (children nested within schools) was undertaken in BiB children where complete data sets from both the mother and child were available, 84 of 2109 children were excluded due to incomplete data (figure 1). This was mainly due to incomplete data on the BPVS which was not recorded in 60 (3%) children. To analyse the effect of VA on literacy, unadjusted analysis was undertaken on BiB children with complete data (n=2025). Subsequent adjustment for demographic and socioeconomic (maternal and child characteristics) including BPVS score to account for cognitive ability was then undertaken. The characteristics included in the statistical analysis were those found to be associated with both educational and visual outcomes in the current literature. Demographic factors were: ethnicity (determined by the mothers’ ethnicity), sex at birth, birth weight, gestational age, language of baseline questionnaire completed by mother, mothers’ place of birth. Socioeconomic factors were: mother in receipt of benefits, level of mothers’ education, mother smoked during pregnancy.^23–28^ The characteristics are detailed in table 1.

**Table 1:** Distribution of characteristics in Born in Bradford (BiB) children with complete data

Characteristic	Mean (SD)
Letter ID score	107.07 (12.5) range 68–143
Visual acuity (logMAR)	0.13 (0.09) range 0.0–0.8
British Picture Vocabulary Score	100.97 (14.47) range 39–160
Mean Spherical Equivalent* (D)	1.07 (0.64) range −2–+9.5
Birth weight (g)	3191 (541) range 680–5180
Gestational age (weeks)	39.14 (1.63) range 27–43
Sex at birth (M:F)	1010:1015
Ethnicity†	n (%)
White British	671 (33.2)
Pakistani	1106 (54.6)
Other	248 (12.2)
Baseline questionnaire language†	n (%)
English	1541 (76)
Other language	484 (24)
UK-born mother†	n (%)
Yes	1177 (58)
No	848 (42)
Receiving benefits†	n (%)
Yes	880 (43.46)
No	1145 (56.54)
Mothers level of education†	n (%)
Low (<5 GCSE equivalent and unknown)	567 (28)
Medium (5 GCSE and A level equivalent)	1050 (52)
High (higher than A level)	408 (20)
Mother smoked in pregnancy†	n (%)
Yes	282 (14)
No	1743 (86)

*n=1893 all other variables n=2025.

†Determined by mothers’ response to the baseline questionnaire.

D, dioptres; F, female; GCSE, general certificate of secondary education; M, male.

The regression analyses were undertaken in three steps: first, demographic factors (listed above) were included in the model; a second model was then run adjusting for the socioeconomic factors (listed above); and finally a fully adjusted model was run adjusting for all demographic and socioeconomic factors and the BPVS score for general cognitive ability. In all these models, 2025 children from 74 schools were included.

Further regression analysis was undertaken to examine the impact of mean spherical equivalent on a subsample with complete data available (n=1893). A sensitivity analysis was also undertaken excluding children unable to carry out letter matching (n=1979). Multilevel analysis was undertaken in order to account for variability between schools; the variance in attainment attributed to differences between schools was calculated to provide a variance partition coefficient for each model. All analyses were carried out using Stata V.13 (StataCorp, College Station, Texas, USA).

## Results

The overall mean (SD) VA for all children (n=11 186) who received vision screening was 0.14 (0.09) logMAR (range 0.0–1.0). In total, 8.7% (977/11 186) of children had a VA worse than 0.2logMAR, 4% (475/11 186) worse than 0.3logMAR and 1.8% (206/11 186) of children demonstrated a VA of worse than 0.4logMAR. There was no clinically significant difference between the BiB and non-BiB children (see online supplementary table S1).

The univariate and adjusted model analyses for the BiB children are shown in table 2. Unadjusted analysis of the BiB children (n=2025) showed that the literacy score was associated with the level of VA. The literacy score reduced by 2.42 points for every one line (0.10logMAR) reduction in VA (95% CI −2.98 to −1.87), p<0.001. When adjusted to account for cognitive ability (BPVS), demographic factors or socioeconomic factors, the impact of VA remained significant and continued to remain statistically significant in the multivariable model after all factors are accounted for with the literacy score reducing by 1.65 (95% CI −2.17 to −1.13), p<0.001 for every one line (0.10logMAR) reduction in VA. The association between VA and literacy remained after a sensitivity analysis was undertaken to investigate the effect of poor literacy by excluding children unable to carry out the letter matching (see online supplementary table S2). Adjustment for mean spherical equivalent made no material difference and by itself was not associated with literacy (p=0.164), it therefore was not included in the model. The variance in attainment attributed to the difference between schools was 9% in the unadjusted model and 12% in the fully adjusted model across 74 schools.

**Table 2:** Associations between Literacy (letter identification score) and visual acuity, British Picture Vocabulary Scale (BPVS), socioeconomic and demographic (child and maternal) factors, n=2025 children, n=74 schools

Factor	Unadjusted Mean difference in literacy scores (95% CI)	Adjusted BPVS Mean difference in literacy scores (95% CI)	Adjusted demographic Mean difference in literacy scores (95% CI)*	Adjusted socioeconomic Mean difference in literacy scores (95% CI)†	Fully adjusted model Mean difference in literacy scores (95% CI)‡
Change in literacy score per one line (0.1log unit) of visual acuity	−2.42 (−2.98 to −1.87) p<0.001	−1.79 (−2.32 to −1.26) p<0.001	−1.72 (−2.24 to −1.19) p<0.001	−1.72 (−2.25 to −1.19) p<0.001	−1.65 (−2.17 to −1.13) p<0.001
Change in literacy score per one unit change in BPVS		0.27 (0.23 to 0.30) p<0.001	0.26 (0.22 to 0.30) p<0.001	0.25 (0.22 to 0.29) p<0.001	0.25 (0.21 to 0 0.28) p<0.001
Ethnicity					
White British			Reference		Reference
Pakistani			0.83 (−0.82 to 2.47) p=0.325		−0.14 (−1.86 to 1.58) p=0.872
Other			3.79 (1.86 to 5.73) p<0.001		2.85 (0.88 to 4.82) p=0.005
Sex at birth					
Male			Reference		Reference
Female			3.01 (2.03 to 3.99) p<0.001		3.06 (2.09 to 4.04) p<0.001
Birth weight (g)			0.001 (0.0001 to 0.002) p=0.028		0.001 (0.0001 to 0.002) p=0.036
Gestational age (weeks)			0.006 (−0.35 to 0.37) p=0.975		−0.01 (−0.37 to 0.34) p=0.937
Questionnaire language					
English			1.78 (0.21 to 3.35) p=0.026		1.61 (3.18 to 0.04) p=0.045
Other language			Reference		Reference
UK born					
Yes			−1.19 (−2.66 to 0.28) p=0.113		−0.97(−0.49 to 2.43) p=0.192
No			Reference		Reference
Receiving benefits					
Yes				−1.05 (−2.06 to −0.03) p=0.043	−1.03 (−2.04 to −0.03) p=0.045
No				Reference	Reference
Level of education					
Low (<5 GCSE equivalent and unknown)				Reference	Reference
Medium (5 GCSE and A level equivalent)				1.14 (−0.024 to 2.3) p=0.055	1.13 (−0.04 to 2.3) p=0.059
High (higher than A level)				3.30 (1.8 to 4.8) p<0.001	3.20 (1.71 to 4.70) p<0.001
Smoked in pregnancy					
Yes				−2.19 (−3.68 to −0.69) p=0.004	−1.82 (−0.25 to −3.39) p=0.023
No				Reference	Reference

*Demographic adjustment includes visual acuity, BPVS, ethnicity, sex at birth, birth weight, gestational age, language of baseline questionnaire, mothers’ place of birth.

†Socioeconomic adjustment includes visual acuity, BPVS, receipt of benefits, level of mothers’ education, mother smoked during pregnancy.

‡Fully adjusted analysis includes all factors: visual acuity, BPVS, ethnicity, sex at birth, birth weight, gestational age, language of baseline questionnaire, mothers’ place of birth, receipt of benefits, level of mothers’ education, mother smoked during pregnancy.

## Discussion

This study is the first to reliably demonstrate that poor VA in young children is associated with reduced early developing literacy. The average receptive vocabulary and slightly above average literacy scores (table 1) of the children indicate that general low achievement does not influence our findings. The mean VA (table 1) of these 4–5-year-old children is similar to previously published normative data^29^; however, our findings indicate a high proportion of children (9%) had reduced VA with 2% classified as visually impaired.^22^ This is likely to impact significantly on their early developing literacy. The Bradford cohort of children demonstrates a higher prevalence of poor presenting VA (defined as worse than 0.3logMAR) compared with that reported elsewhere^19^ ^21^ ^23^ ^30^ (table 3).

**Table 3:** Comparison of studies reporting prevalence of poor visual acuity (worse than 0.30logMAR)

Author	Community	Age (years)	Number of participants	Prevalence (%)
Robaei *et al*^19^	Australia	6–7	1738	0.9
Friedman *et al*^21^	USA	2.5–5.5	1714	1.5
Williams *et al*^23^ O'Donoghue *et al*^30^	Bristol, UK Northern Ireland, UK	7 6–7	7825 392	0.6 1.5
Bruce *et al*	Bradford, UK	4–5	11 186	4.0

For the majority of children in Bradford, vision screening at school entry is their first assessment of visual status with few having had any previous treatment; this is likely to account for the increased prevalence observed. In this study, 2% of children were wearing glasses at vision screening, similar to that found in an urban population of children aged 30–71 months in the USA (1.7%),^21^ but substantially lower than the 4.4% of children aged 6 years in Australia.^19^ Another UK cohort study^23^ reported 0.6% prevalence of poor presenting VA at the age of 7 years; however, 3% of the children in their sample had undergone previous treatment. The prevalence reported in the US study was 1.2% in white children and 1.8% in black children.^21^ In our study, 2.7% of white British, 5.2% of Pakistani children and 2.8% of other ethnicities had VA worse than 0.3 logMAR. In both studies, the differences in VA between the ethnic groups were not statistically significant.

It has been shown that children from socioeconomically deprived households have an increased prevalence of vision problems,^31^ ^32^ which may in part be due to inequality in accessing health services.^33^ The Bradford vision screening programme covers 97% of children^14^ and therefore does not exclude children from the lower socioeconomic areas. The high levels of deprivation in the city may help explain the higher prevalence level of poor VA. Educational attainment is multifactorial and influenced by social disadvantage and demographic factors, differences manifest early and are demonstrable through gaps in literacy achievement.^27^ ^28^ Factors known to be associated with educational outcome such as socioeconomic status,^28^ ^34^ gender^35^ and mothers’ education^36^ were also shown in this study to impact on literacy (table 2). There was no difference between the literacy scores of the white British and the Pakistani children; however, there was a positive association between literacy and VA for children in the ‘other’ ethnic category. A third of children in this category had mothers with high educational attainment and this may help explain the association. The association between the level of VA and literacy remains significant after adjustment for socioeconomic and demographic factors (table 2).

Low degrees of refractive error, in particular, hyperopia are normally reported in young children.^37^ A few studies have found that low degrees of uncorrected hyperopia in young children have an impact on literacy.^38^ ^39^ Non-cycloplegic autorefraction was used in this study to provide an indication of refractive status. Commonly non-cycloplegic refraction underestimates the level of hyperopia present in young children,^40^ autorefraction using the Welch-Allyn has however been shown to have a small hyperopic bias^41^ which could have increased the reported mean spherical equivalent of the Bradford population (table 1). All children who failed their vision screening assessment were referred for a cycloplegic examination to confirm refractive error; an ongoing longitudinal study of these children will examine the results. In this study, our analysis demonstrates an association between literacy and VA but not refractive error.

A small number of population-based studies have examined the impact of VA on educational outcome. A US study evaluating the effect of visual function on academic performance (children aged 6–9 years) found no association. However, the key indicator of academic performance (Metropolitan Readiness Test) was not available for a large proportion of the children and a proxy measure of attainment was used, neither did the study take into account the effects of potential confounding factors.^42^ Retrospective analysis of the 1958 British birth cohort reporting outcomes at age 11 years found no association between unilateral amblyopia and educational, health and social outcomes; however, participants with bilateral visual loss were excluded from the study.^43^ A large cohort study in Singapore reported no effect of presenting VA on academic school performance,^44^ but the Singapore cohort of children at age 9–10 years only included a small number of children with poor vision which reduced the power of the study to detect any significant association.

Our paper reports the largest population-based study which explores the impact of VA on literacy and has a number of strengths. The cohort is set in a multiethnic population, and the use of data linkage has allowed us to undertake rigorous analysis taking into account the effect of potential confounding factors. However, there are limitations, 2929 out of 5836 (50%) of BiB children had received a literacy test at the time of data linkage; this reduced the number of children (n=2025) who had complete data sets and may compromise the representativeness of the sample. However, comparison of the BiB children (n=2025) with complete data demonstrated a similar percentage of children within each quintile of the Index of Multiple Deprivation and is comparable to the complete BiB cohort of children (n=13 773).^10^ The prevalence of poor vision in this cohort of children (n=2025) is also similar to all Bradford children (n=11 186; see online supplementary table S1). As a proxy indicator for English as a second language, we used the language in which the baseline questionnaire was completed by the mother during pregnancy. Although all children are taught in school in English, this may not be the primary language of choice at home; this information was not available.

The study has the inherent limitations of a cross-sectional design, which reduces our ability to confidently infer causality. However, it is unlikely that poor literacy resulted in poor performance in the vision test; the majority of children (98%) performed the recommended age appropriate vision test and the association between vision and literacy remained after excluding children unable to accomplish the letter matching. In addition, if indeed poor literacy causes poor vision we would expect that those children with specific reading difficulties (dyslexia) would demonstrate reduced VA. In a recent study four out of five children with reading difficulties demonstrated normal visual function.^45^

By linking the clinical data set from the population-based vision screening programme with epidemiological data from a large birth cohort study, along with the baseline literacy assessments, this is the first multiethnic population-based study to have the statistical power to take into account the multiple factors that are known to impact on educational outcomes. Our results demonstrate a significant association between VA and early literacy. In a population with a high prevalence of reduced vision, this has important implications for children's future educational outcomes. The reduction in the literacy score by around 2% for every line of vision reduction is important in a population where there are poor levels of vision on school entry. This study strengthens the argument for a national vision screening programme. The impact of such a programme will depend on the degree to which detection of reduced vision at age 4–5 years results in effective intervention to improve vision and the impact this has on health, educational and social outcomes. Further research is required to determine the extent to which children with poor vision access treatment and the impact of such treatment not only on levels of vision but also on their educational attainment.
